# The influence of health literacy on the timely diagnosis of symptomatic cancer: A systematic review

**DOI:** 10.1111/ecc.12920

**Published:** 2018-10-16

**Authors:** Elka Humphrys, Jenni Burt, Greg Rubin, Jon D. Emery, Fiona M. Walter

**Affiliations:** ^1^ Primary Care Unit, Department of Public Health and Primary Care University of Cambridge Cambridge UK; ^2^ Cambridge Centre for Health Services Research Department of Public Health and Primary Care University of Cambridge Cambridge UK; ^3^ Institute of Health and Society Sir James Spence Institute, Newcastle University Newcastle upon Tyne UK; ^4^ Department of General Practice and Centre for Cancer Research University of Melbourne, Victorian Comprehensive Cancer Centre Melbourne Victoria Australia

**Keywords:** cancer, health literacy, timely diagnosis, systematic review

## Abstract

Low health literacy has been associated with poor cancer screening uptake, difficulty in making treatment choices and reduced quality of life following a cancer diagnosis, yet it is unclear whether and how health literacy influences the pathway to diagnosis for patients with cancer symptoms. This systematic review aimed to evaluate the influence of health literacy on the timely diagnosis of symptomatic cancer. Literature was searched between January 1990 and May 2017 using MEDLINE, Embase, Scopus, ASSIA, CINAHL and PsycINFO. Only three papers met the inclusion criteria. These reported two qualitative studies and one quantitative, with adult patients diagnosed with gastrointestinal (colon, rectum and pancreas), cervical and breast cancer. The definition and assessment of health literacy varied between the studies, as did the descriptions of the pathway to diagnosis. Due to the methodological weaknesses identified, the conclusions are limited; however, the studies did highlight important considerations in the definition and measurement of health literacy. Further research is required that clearly defines health literacy and follows the principles of the Aarhus Statement to assess the influence of health literacy on the pathway to cancer diagnosis. The protocol for this review was registered with PROSPERO (CRD42016048917).

## 1 INTRODUCTION

Public Health England (2015) has described health literacy as the “bridge between people and health settings,” reflecting how patients access, understand, evaluate and use healthcare information and navigate the services available to them. This multi‐dimensional concept reflects how our understanding of health literacy has developed since the 1990s, when the first widely used instruments to measure health literacy were published, focusing on comprehension and numeracy skills (Baker, Williams, Parker, Gazmararian, & Nurss, 1999; Davis et al., 1991). In recent years, studies have explored health literacy in relation to cancer and found that low health literacy is associated with poor cancer screening uptake (Oldach & Katz, 2014; von Wagner, Semmler, Good, & Wardle, 2009), difficulty in making treatment decisions once diagnosed (Amalraj, Starkweather, Nguyen, & Naeim, 2009; Koay, Schofield, & Jefford, 2012) and reduced quality of life in cancer patients (Husson, Mols, Fransen, Poll‐Franse, & Ezendam, 2015; Song et al., 2012). Oldach and Katz’s (2014) review identified 14 studies of cancer screening (colorectal, breast, cervical and prostate) and concluded that there was a trend towards low screening rates with low health literacy. This could be influenced by the association of low health literacy with low knowledge of cervical cancer screening (Lindau et al., 2002), or the burden on patients with low health literacy to read and understand written information in relation to colorectal cancer screening (von Wagner et al., 2009). Understanding information, both written and oral, can also be difficult for those with low health literacy faced with complex treatment options, thereby limiting engagement and participation in shared decision‐making (Amalraj et al., 2009). Difficulties in communication may also act as a barrier for accessing support services, possibly contributing to worse mental health outcomes as seen for low health literate men newly diagnosed with prostate cancer (Song et al., 2012). While this suggests that low health literacy may affect cancer screening and treatment pathways, it remains unclear how health literacy may influence the timely diagnosis of cancer in symptomatic patients.

Promoting timely diagnosis has been a priority in improving outcomes for cancer patients since the launch of the National Awareness and Early Diagnosis Initiative (NAEDI, 2008) in the UK. Key findings have identified a significant variation in the time to diagnosis across cancers, from symptom onset to first presentation in primary care, subsequent referral and cancer diagnosis (Din et al., 2015; Lyratzopoulos et al., 2015). Frameworks such as the Categorisation of Delay model (Olesen, Hansen, & Vedsted, 2009) and the Model of Pathways to Treatment (Scott, Walter, Webster, Sutton, & Emery, 2013; Walter, Webster, Scott, & Emery, 2012) are useful for describing the intervals along the pathway to diagnosis, while the Aarhus Statement suggested ways to improve the design and reporting of studies (Weller et al., 2012). Outcomes could be improved by decreasing the time between a patient first noticing a potential cancer symptom and seeking help (appraisal and help‐seeking, or patient interval), and reducing the time between initial consultation with a healthcare professional, referral to secondary care and diagnosis (diagnostic, or primary and secondary care interval). In considering the pathway to diagnosis, it is possible that an individual's health literacy may influence timely diagnosis through a person's ability to access and understand cancer symptom information, appraise the information in relation to their own bodily changes and navigate the healthcare system; presenting to a healthcare practitioner and accessing the specialist care required to receive a diagnosis.

In this systematic review, we therefore aimed to evaluate the impact of health literacy on the timely diagnosis of symptomatic cancer.

## 2 METHODS

### 2.1 Search strategy

We searched peer‐reviewed literature published worldwide from 1 January 1990 to 19 May 2017. The search was limited to 1990 onwards as health literacy is a relatively new field, with the number of studies expanding following the publication in 1991 of the first widely used health literacy instrument, the Rapid Estimate of Adult Literacy in Medicine (REALM) (Davis et al., 1991). Six bibliographic databases were searched, with the strategy developed and run in MEDLINE (Table 1) and adapted for Embase, Scopus, ASSIA, CINAHL and PsycINFO. Further articles were identified from the reference lists of included studies, other work published by included authors and running a forward citation search in Scopus. Grey literature was excluded as a prior scoping exercise had not identified other relevant information sources. Citations were imported and managed in EndNote X7.

**Table 1:** Search strategy for MEDLINE^a^

Search	Query
1	“Cancer*”.mp
2	“Tumour*”.mp
3	“Tumor*”.mp
4	“Malignan*”.mp
5	“Neoplasm*”.mp
6	exp Neoplasm/
**7**	**Or/1–6**
8	exp Health Literacy/
9	“Health Literacy”.mp
10	“Health Literate”.mp
11	“Health Literacies”.mp
12	“Literacy”.mp
13	“Literate”.mp
14	“Literacies”.mp
15	“Cancer literacy”.mp
16	“Cancer literate”.mp
17	“Numeracy”.mp
18	“Numerate”.mp
**19**	**Or/8–18**
**20**	**7 AND 19**
21	“Systematic review”.ti
**22**	**20 NOT 21**
**23**	**Limit 22 to yr="1990 ‐Current"**

Bold is used to identify where individual searches have been grouped together.

a Also used for Embase.

### 2.2 Inclusion and exclusion criteria

Studies published in any language were included where they focused on adult patients (aged 18 years and older) with a primary diagnosis of any cancer and explored the influence of health literacy (or literacy/numeracy related to health yet not termed as “health literacy”) in relation to the time to diagnosis of symptomatic cancer. This included studies evaluating the total time to diagnosis, from symptom onset to diagnosis, or focusing on one or more intervals along the pathway: appraisal, help‐seeking or diagnostic intervals. Systematic reviews, reviews, editorials and letters were excluded, along with studies reporting time to diagnosis without health literacy, or vice versa, or studies focusing on recurrent cancers, cancer incidence, survival and mortality, risk factors, genetics, screening and prevention, or assessing the validity of referral decisions.

### 2.3 Study selection

Following removal of duplicate references, EH screened the titles and abstracts against the inclusion and exclusion criteria, with a random sample (10% of the total) assessed by FMW and JB to confirm agreement. The full text was obtained for all studies identified as potentially relevant to the review. Three reviewers (EH, FMW and JB) screened all the full‐text articles to identify studies for inclusion in the review.

### 2.4 Data extraction

Data were extracted by EH from each of the included studies: study type, recruitment setting, data collection details, participant characteristics, patient pathway/interval data as defined within the study, health literacy data including the definition and health literacy instrument used (if any), and the findings in relation to time to diagnosis. The extracted data were reviewed by FMW and JB to confirm completeness.

### 2.5 Quality assessment

Quality of the studies was assessed by EH and reviewed by FMW and JB, using the Joanna Briggs Institute Critical Appraisal Tools (Joanna Briggs Institute, 2016). The aim of the quality assessment was to determine the validity of the results based on the design, methods, analysis and conclusions of each study and to assess the relative contribution of each study to the review.

### 2.6 Protocol registration and reporting

Prior to starting the review, the protocol was registered with PROSPERO (CRD42016048917), an international prospective register of systematic reviews. The review is reported based on the guidelines proposed by the PRISMA Statement: Preferred Reporting Items for Systematic Reviews and Meta‐Analyses (Moher, Liberati, Tetzlaff, Altman, & The, 2009).

## 3 RESULTS

### 3.1 Study selection

**Figure 1:**
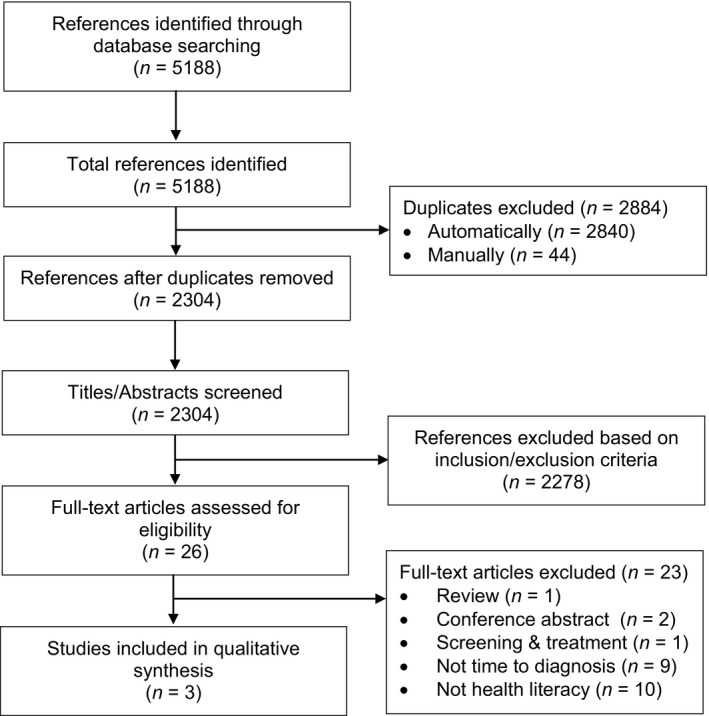
PRISMA flow diagram of the systematic review process

The search identified 5,188 citations, and after removing duplicates, 2,304 titles and abstracts were screened against the inclusion and exclusion criteria to identify 26 potentially includable studies (Figure 1). Following full‐text assessment, three studies qualified for inclusion. The reference lists of the included studies and all other publications by the authors of those studies were reviewed. The forward citation search in Scopus did not identify any additional relevant studies.

### 3.2 Study characteristics

The three included studies were set in Japan, the USA and Egypt, and included patients diagnosed with gastrointestinal (colon, rectum and pancreas), cervical or breast cancer (Table 2). Two studies used qualitative methods, while the third reported a survey. The number of participants ranged from 10 to 37 and the overall sample was predominately female (92%). The studies set in the USA and Egypt were published in English, while the study set in Japan was published in Japanese and was therefore professionally translated. Due to the heterogeneity of studies, it was not possible to synthesise the findings; therefore, we chose to use a descriptive approach to analyse and report the findings.

**Table 2:** Study and participant characteristics

Author (year)	Country	Language	Setting	Cancer	Sample size	Age range (years)	Female (*n*; %)	Study type	Recruitment
Nakagami and Akashi (2010)	Japan	Japanese	Inpatient	Colon, rectum, pancreas	10	Early 40s–late 70s^a^	5; 50%	Qualitative	Prior to surgery
Tecu and Potter (2012)	USA	English	Outpatient Tumour Clinic	Cervical	37	25–62	37; 100%	Survey	First chemotherapy treatment
McEwan et al. (2014)	Egypt	English	Cancer Treatment Centre^b^	Breast	15	29–60	15; 100%	Qualitative	Post‐treatment^c^

a Age as defined within the study. The precise age of the participants was not stated.

b Setting defined within the previous associated quantitative study (Corbex, 2010).

c Timing of recruitment post‐treatment was not specified.

### 3.3 Quality of included studies

The studies were assessed based on methodological quality and conceptual clarity in relation to definitions of “time to diagnosis” and “health literacy.”

#### 3.3.1 Methodological quality

Table 3 summarises the methodological quality of the studies, which ranged from poor to adequate. Study details such as setting, sampling strategy and exclusion criteria were poorly described, with only one study fully describing the participant inclusion criteria (Nakagami & Akashi, 2010). The small sample sizes impacted the analysis, with Nakagami and Akashi unable to reach theoretical saturation based on the grounded theory approach used (Strauss & Corbin, 1998). From the reporting of the qualitative studies, it was also unclear whether the data accurately reflected the voices of most participants or a minority (McEwan, Underwood, & Corbex, 2014; Nakagami & Akashi, 2010). While acknowledging the limitations in the study design and conclusions, studies were not excluded based on the quality assessment alone.

**Table 3:**
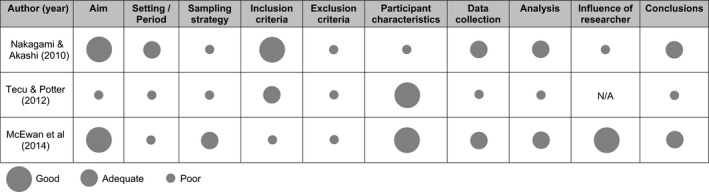
Methodological quality of included papers

#### 3.3.2 Conceptual clarity

##### Time to diagnosis

All the studies aimed to explore the diagnostic pathway from the patients’ perspective, from symptom onset to diagnosis or start of treatment. Table 4 summarises the definition(s) used by the authors to describe the intervals along the pathway, and the corresponding interval defined by the Model of Pathways to Treatment (Scott et al., 2013; Walter et al., 2012) and the Categorisation of Delay model (Olesen et al., 2009). The definitions of the intervals within the studies were poor with only one study providing a definition of symptom onset and none defining the date of diagnosis. It was therefore unclear as to the time points defining the beginning and end of the intervals and the way the date of diagnosis was identified. This is an important issue raised by the Aarhus Statement, which highlighted that there was little consistency in the definition and measurement of intervals and key time points along the patient pathway (Weller et al., 2012).

**Table 4:** Definitions of intervals used in the included studies

Author (year)	Author's interval definition	Model of Pathways to Treatment	Categorisation of Delay model
Nakagami and Akashi (2010)	Initial symptom detection to cancer diagnosis	Appraisal, Help‐seeking, Diagnostic	Patient, Primary Care, Secondary Care (Diagnostic)
Tecu and Potter (2012)	Symptom onset to first presentation	Appraisal, Help‐seeking	Patient
	Symptom onset to receipt of first treatment	Appraisal, Help‐seeking, Diagnostic, Pre‐treatment a	Total (Patient, Diagnostic, Treatment)
McEwan et al ( 2014)	Symptom discovery to initial medical consultation	Appraisal, Help‐seeking	Patient
	Initial medical consultation to diagnosis	Diagnostic	Primary Care, Secondary Care (Diagnostic)
	Diagnosis to initiation of treatment	Pre‐treatment a	Treatment a

a The pre‐treatment/treatment interval is the final interval within each model ‐ not the focus of this review.

The study by Tecu and Potter was the only one to provide details on interval lengths as reported by the patients. Based on these data, the average time from symptom onset to first presentation (i.e. patient, or appraisal and help‐seeking interval) was 5.26 months (*SD* unknown), while symptom onset to treatment onset (i.e. total interval) was 8.82 months (*SD* ± 11.41, range 1–48 months). The two qualitative studies did not seek to quantify the time from symptom onset to diagnosis, although patients’ narratives were used to demonstrate how the time to diagnosis may be influenced (McEwan et al., 2014; Nakagami & Akashi, 2010). Participants included in the qualitative study by McEwan et al. were sampled from a prior quantitative study assessing the time to diagnosis in breast cancer patients. Based on the quantitative study data, 12 of the 15 participants selected for interview had experienced a delay in the appraisal, help‐seeking, diagnostic or pre‐treatment interval, although the authors did not state what constituted a “delay.”

##### Health literacy

The definition and assessment of health literacy varied between the three included studies (Table 5). Two defined the concept of health literacy based on previous definitions, with Tecu and Potter referencing the US Department of Health and Human Services (Healthy People, 2000), and Nakagami and Akashi referring to seven definitions, with a focus on three (Mancuso, 2008; Murata, Arakita, & Shirai, 2006; Nutbeam, 2000). McEwan et al. used their own definition, describing health literacy in relation to symptom interpretation and knowledge networks. However, they also used a conceptual framework, the “social ecological model” (Scheidner, 2006), to analyse and present their data, which extended the definition of health literacy to include patient beliefs around risk factors.

**Table 5:** Health literacy definitions and assessment used in the studies

Author (year)	Health literacy		Assessment
	Literature referenced	Definition in the literature	
Nakagami and Akashi (2010)	Nutbeam^a^ (Nutbeam, 2000) Speros^b^ (Speros, 2005) Mancuso^c^ (Mancuso, 2008)	Abbreviated definition as stated in the paper: “‘Ability to acquire information relating to illness, medicine and health’ (information about medicine and health), ‘ability to compute’, ‘ability to read medical and health information’, ‘ability to understand medical and health information’, ‘ability to take the role of the patient’, and ‘ability to take appropriate decisions and to evaluate’”	Explored via interviews
	Murata (Murata et al., 2006)	“‘Reading and writing and computation’, ‘information acquisition’, ‘perception, cognition and understanding’. ‘analysis, selection and evaluation’, ‘action’, response’ and ‘provision to others’”	
	World Health Organisation (Nutbeam, 1998	The cognitive and social skills which determine the motivation and ability of individuals to gain access to understand and use information in ways which promote and maintain good health.	
	American Medical Association (Ad Hoc Committee on Health Literacy, 1999)	The constellation of skills, including the ability to perform basic reading and numeral tasks required to function in the healthcare environment	
	US Department of Health and Human Services (Healthy People, 2000)	The degree to which individuals have the capacity to obtain, process, and understand basic health information and services needed to make appropriate health decisions	
Tecu and Potter (2012)	US Department of Health and Human Services (Healthy People, 2000)	As above	REALM‐SF
	National Network of Libraries of Medicine (NNLM, 2011)	Does not provide a unique definition of health literacy. References the US Department of Health and Human Services definition	
McEwan et al (2014)	None	Risk factors, symptom interpretation and knowledge networks (authors’ definition). ‘Social ecological model’ (Scheidner,^2006^)	Explored via interviews

a Original definition from Nutbeam (2000): “The personal, cognitive and social skills which determine the ability of individuals to gain access to, understand, and use information to promote and maintain good health.”

b Speros 2005 does not provide a unique definition of health literacy and instead references the definitions provided by the World Health Organisation, American Medical Association, and US Department of Health and Human Services as above.

c Original definition from Mancuso (2008): “A process that evolves over one's lifetime and encompasses the attributes of capacity, comprehension, and communication. The attributes of health literacy are integrated within and preceded by the skills, strategies, and abilities embedded within the competencies needed to attain health literacy.”

To assess health literacy, Tecu and Potter used the short‐form of the REALM (REALM‐SF) (Davis et al., 1993), a validated instrument designed to assess pronunciation of medical words. It has been widely used in health literacy research but is primarily an assessment of comprehension rather than an assessment of ability to obtain and use health information. From the analysis, the assessment of the REALM‐SF score in relation to time to presentation was limited (Tecu & Potter, 2012). The two qualitative studies explored health literacy via interviews with patients and in relation to the definitions of health literacy as proposed within each study.

### 3.4 Influence of health literacy on time to diagnosis

The two qualitative studies took different approaches to exploring health literacy, with Nakagami and Akashi focusing their analysis on how aspects of health literacy influenced the pathway to diagnosis in symptomatic and asymptomatic patients, while McEwan et al. explored many factors influencing the pathway, with health literacy being one of these.

Both studies discussed health literacy in relation to awareness and knowledge, with Nakagami and Akashi also considering symptom interpretation and how this influenced the appraisal and help‐seeking intervals of the pathway to diagnosis. Where the patient attributed a benign cause to their symptoms, their action was to “watch and wait,” often resulting in a lengthening of the appraisal interval. Nakagami and Akashi referred to this as forming a “disease hypothesis,” where health literacy skills were used to process symptom information and consider prior experience and knowledge, to understand and evaluate symptoms. When symptoms persisted or worsened, the patient would often obtain further information from other sources and re‐evaluate their symptoms in the context of this new information. Nakagami and Akashi also considered the role of social networks in the pathway to diagnosis, an important aspect of current multi‐factorial models of health literacy (Osborne, Batterham, Elsworth, Hawkins, & Buchbinder, 2013), and found that knowledge obtained from family, friends or neighbours could often prompt help‐seeking. When considering risk factor knowledge, participants in the study by McEwan et al. were able to identify multiple possible risk factors, although many believed that being angry or upset had caused their cancer. Where participants did not think they were at risk of cancer, time to presentation was longer and so the authors stated that “poorer health literacy increased delays” (McEwan et al., 2014).

Focusing on the diagnostic interval, Nakagami and Akashi found that it was lengthened where an alternative diagnosis was suggested or the patient was monitored or given medication for their symptoms. In these instances, patients had to re‐start the process of understanding, evaluating and acting on their symptoms, which relied on their health literacy ability.

In contrast to the qualitative studies, the survey study conducted by Tecu and Potter quantified the time to diagnosis and explored health literacy using the REALM‐SF. The mean REALM‐SF score was 60.08 (*SD* 12.63, range 0–66), with eight (22%) women scoring 54 or less and demonstrating low health literacy. The authors stated that no statistically significant correlations were found between the REALM‐SF scores and the patient interval (time from symptom onset to first presentation) across the whole cohort; however, they did not provide data to substantiate this. A sub‐group analysis of the eight women with low health literacy found that four had a patient interval of 6–12 months; although, patient intervals were not reported for the 29 women who scored >54 on the REALM‐SF. The survey also explored symptom experience, knowledge and help‐seeking behaviours, yet presented these as descriptive statistics without any analysis of how these factors may correlate with health literacy or the patient interval.

## 4 DISCUSSION

This systematic review sought to explore how health literacy can influence the patient's pathway to diagnosis with cancer, as health literacy may affect a patient's ability to access and understand cancer symptom information, appraise the information in relation to bodily changes and navigate the healthcare system to access the specialist care required to obtain a diagnosis. Although the search strategy was broad and undertaken across multiple databases, only three studies, all methodologically poor, met the inclusion criteria. There is an important gap in the literature when considering the role of health literacy in timely diagnosis of cancer.

The included studies used three very different approaches to assess health literacy, which reflects the current challenge of health literacy research in respect to definitions and measurement. Sorensen et al. (2012) conducted a systematic review and identified 17 definitions of health literacy, while Jordan et al. and Altin et al. identified 36 instruments (19 and 17 respectively) to measure health literacy in a general population (i.e. not disease specific) published between 1990 and 2008 (Jordan, Osborne, & Buchbinder, 2011), and January 2009 and April 2013 (Altin, Finke, Kautz‐Freimuth, & Stock, 2014). A systematic review of health literacy in adolescents and young adults discussed how current literature focuses on quantifying health literacy, usually assessing abilities as adequate or inadequate, whereas a qualitative approach could help to understand the wider concept and factors influencing health literacy ability (Sansom‐Daly et al., 2016). Tecu and Potter opted to use the REALM‐SF to measure health literacy, yet concluded that a more comprehensive tool would have been more appropriate to the study aims and their definition of health literacy as based on the US Department of Health and Human Services (Healthy People, 2000). This definition focuses on how individuals obtain, process and use health information and services, whereas the REALM‐SF is a test of comprehension and is therefore not intended to measure application of health information. McEwan et al. proposed a very narrow definition in their qualitative study, yet indirectly assessed other aspects of health literacy, in the context of current definitions (Osborne et al., 2013), such as social networks, patient‐provider communication and navigation of the healthcare system. As McEwan et al. did not specifically relate these findings to health literacy, these results were outside the scope of this review. In comparison, Nakagami and Akashi used a clear definition of health literacy, yet were limited in their conclusions as the sample was inadequate to reach theoretical saturation and included five asymptomatic patients, which did not reflect the aim of their study. The asymptomatic patients were diagnosed following routine hospital‐based check‐ups for other conditions or as part of a local health check for older patients. The results have not been discussed within this review as the focus was diagnosis of symptomatic cancer. Future studies would benefit from describing health literacy in relation to current definitions, and using a validated instrument as a framework for exploring health literacy when using qualitative methods, or to quantify health literacy using survey methods. A suitable instrument for both qualitative and quantitative methods could be the Health Literacy Questionnaire (HLQ), published in 2013 (Osborne et al., 2013). The HLQ is self‐administered (completed by the participant) and assesses the multi‐dimensional concept of health literacy with 44 items across nine domains (4–6 items each). The instrument can be used in full or by selecting domains to assess specific aspects of health literacy, as each domain is scored individually (range 1–5) and can therefore be used independently. This provides a flexible approach suitable for use with multiple methods to explore health literacy along the pathway to diagnosis for symptomatic cancer.

### 4.1 Health literacy and stage at diagnosis

Two of the 23 studies excluded from the review following full‐text assessment used validated measurement tools to assess health literacy in relation to stage at diagnosis (Bennett et al., 1998; Busch, Martin, DeWalt, & Sandler, 2015). Advanced stage disease is a major contributory factor to the poor survival outcomes across many cancers in the UK in comparison with Europe (Robb et al., 2009), and there are major efforts being made internationally to detect cancer at an earlier stage (Lyratzopoulos et al., 2012). A recent systematic review demonstrated the association between time to diagnosis and disease stage for some common cancers with equivocal findings for less common cancers (Neal et al., 2015). As it is unclear whether stage is an accurate indicator of time to diagnosis, the studies exploring health literacy and stage at diagnosis were excluded from the current systematic review.

### 4.2 Strengths and limitations of the review

In searching a wide range of databases with a broad search strategy, we are confident that we identified all articles that aimed to explore health literacy on the time to diagnosis of cancer. However, definitions of health literacy vary considerably, as seen in the study by McEwan et al. where aspects of health literacy were explored without defining them as “health literacy.” In this review, we specifically searched for “health literacy” or variations of the term and therefore other studies exploring time to diagnosis of cancer, particularly using qualitative methodology, could have investigated areas that are linked to health literacy yet not recognised them within this context and therefore these papers would not have been identified within the review.

A further review investigating qualitative time to diagnosis research and evaluating the results in respect to a recent multi‐dimensional definition of health literacy may now be needed.

## 5 CONCLUSION

Due to the few studies identified from the systematic search, and their methodological weakness and relatively poor quality, it was not possible to fully evaluate the influence of health literacy on the timely diagnosis of symptomatic cancer. However, the studies provide a starting point for research within this area and identify important aspects that need to be addressed in future research. When exploring diagnostic routes for cancer, researchers should be guided by the Aarhus Statement and underpin their research with a conceptual framework and clear definitions. Where health literacy is explored, researchers should also be aware of the range of health literacy definitions and assessment tools currently in use, and how these could relate to their research. Again, they would be advised to choose a definition best suited to their research area and to reflect on this and the aim of the research when choosing an appropriate instrument or method for exploring health literacy.

Reducing the patient interval is important for earlier diagnosis of cancer and it is possible that health literacy may influence the pathway, which could have important implications for developing targeted awareness campaigns for recognition of cancer symptoms and to prompt timely help‐seeking, as well as informing GP‐patient communication strategies. Research exploring the time to diagnosis should also consider the relation of health literacy to other factors affecting the pathway. In conclusion, further research is required that clearly defines health literacy and adheres to the principles of the Aarhus Statement to assess the influence of health literacy on the timely diagnosis of symptomatic cancer.

## CONFLICT OF INTEREST

None.

## AUTHOR CONTRIBUTIONS

All authors contributed to the design of the study and analysis of data, and were involved in drafting and revising the manuscript.
